# Global patterns and demographic drivers of hearing loss attributable to otitis media, 1990–2023

**DOI:** 10.1371/journal.pone.0356787

**Published:** 2026-08-28

**Authors:** Yubin Xue, Hongwei Ma, Lin Yang, Qiang Liu

**Affiliations:** 1 Department of Otorhinolaryngology, Beijing Tiantan Hospital, Capital Medical University, Beijing, China; 2 Department of Science and Education, The First People’s Hospital of Shizuishan, Ningxia Medical University, Shizuishan, Ningxia, China; Manipal Academy of Higher Education, INDIA

## Abstract

**Background:**

Otitis media is a common childhood condition and a leading cause of preventable hearing loss worldwide. Although global hearing loss has been widely studied, recent and comparable estimates focusing specifically on hearing loss attributable to otitis media across age, sex, development level, and countries remain limited. Updated assessments are needed to characterise long-term patterns and demographic drivers.

**Methods:**

We analysed Global Burden of Disease 2023 estimates at the global level, across four Socio-demographic Index (SDI) groups, and among selected countries with available country-level estimates from 1990 to 2023. Age-standardised, age-specific, and sex-specific estimates were examined. Temporal trends were assessed using annual percentage change (APC) derived from log-linear regression models. A stepwise decomposition analysis quantified the contributions of population growth, population ageing, and changes in age-specific YLD rates to overall YLD change. All analyses were conducted using R version 4.3.2.

**Results:**

Globally, age-standardised prevalence declined modestly between 1990 and 2023, with consistently higher levels observed in low and low-middle SDI settings. In 2023, prevalence peaked among children aged 5–14 years and declined steadily with increasing age, reaching the lowest levels among adults aged ≥70 years. Males had higher prevalence than females across most settings. Mild hearing loss accounted for nearly four-fifths of the global age-standardised burden, consistent with the severity structure of otitis media–attributable hearing loss in the Global Burden of Disease framework. No country showed a statistically significant increase in APC; most exhibited stable or declining trends, with faster decreases observed in several middle- and high-middle SDI countries. Decomposition analysis indicated that population growth was the primary contributor to increasing global YLDs, while declines in age-specific YLD rates partially offset demographic pressures in many regions.

**Conclusions:**

Hearing loss attributable to otitis media remains an unevenly distributed global health burden, particularly among school-aged children and populations in lower-SDI settings. These findings describe long-term trends and demographic drivers relevant to planning prevention and ear and hearing care services.

## Introduction

Otitis media is one of the most common childhood infections worldwide and a major cause of preventable hearing loss. While acute episodes are often self-limiting, recurrent or chronic disease can lead to persistent conductive hearing loss, delays in speech and language development, and substantial long-term social and educational consequences [1–3]. Global evidence indicates that the burden of otitis media and its hearing-related outcomes is closely influenced by socioeconomic conditions, household crowding, nutritional status, and access to vaccination and primary ear care services [4,5].

The WHO World Report on Hearing (2021) and the Global Burden of Disease (GBD) 2019 Hearing Loss Collaborators both note that hearing loss remains disproportionately concentrated in low- and low-middle SDI settings, where limited access to early diagnosis, newborn screening, and timely treatment constrains progress [6,7]. Although pneumococcal conjugate vaccination (PCV) and Haemophilus influenzae type b (Hib) immunisation reduce otitis media incidence [2,8], substantial regional variation in vaccine coverage persists, as shown in global analyses [9], contributing to ongoing inequalities.

Previous global analyses have described long-term incidence patterns and disability associated with otitis media [1,10], but updated assessments focusing specifically on hearing loss attributable to otitis media are lacking. No published study has comprehensively examined severity distribution, age–sex patterns, temporal trends, demographic drivers, or cross-country and Socio-demographic Index (SDI)-related disparities using the most recent GBD 2023 estimates. Recent work suggests that demographic change—particularly rapid population growth—continues to shape the global burden of ear and hearing disorders [11], reinforcing the need for updated estimates to guide prevention, early detection, and integrated hearing care planning.

This study provides an updated global assessment of hearing loss attributable to otitis media from 1990 to 2023 using the latest GBD 2023 analytical framework.

## Materials and methods

This study used data from the Global Burden of Disease (GBD) 2023 study to estimate the global burden and selected country-level patterns of hearing loss attributable to otitis media from 1990 to 2023. Seven CSV files were downloaded from the Institute for Health Metrics and Evaluation (IHME) GBD Results Tool and merged into a unified dataset. The analytical code and derived outputs used for data processing and statistical analyses are publicly available in a Zenodo repository [12]. Global and SDI-level estimates were analysed using aggregated GBD locations, whereas country-level analyses were restricted to locations available in the downloaded dataset.

Extracted variables included prevalence, years lived with disability (YLDs), severity categories (mild and moderate hearing loss), age group, sex, location, SDI, and calendar year. Age groups were defined as <5 years, 5–14 years, 15–49 years, 50–69 years, and ≥70 years, together with age-standardised estimates. These age groups followed predefined GBD classifications and were not further subdivided because age-specific estimates were provided according to the standard GBD age structure. Country names were harmonised to ensure consistency across datasets.

All analyses followed the GBD 2023 cause–sequela framework for otitis media–attributable hearing loss and adhered to the Guidelines for Accurate and Transparent Health Estimates Reporting (GATHER) recommendations [13]. Prevalence was defined as the number of otitis media–attributable hearing loss cases per 100 000 population. YLDs were calculated within the GBD framework by combining prevalence with severity-specific disability weights. Analyses were restricted to mild and moderate hearing loss, the only severity categories defined for otitis media–attributable hearing loss in the GBD framework.

SDI was used to characterise development level. Categories followed the standard classification provided by the IHME GBD 2023 framework, including low, low-middle, middle, and high-middle groups. Age-standardised prevalence and YLD rates were summarised globally and by SDI group. Age- and sex-specific patterns were examined for 2023 in six populous and regionally representative countries. Severity composition was assessed by calculating the proportion of mild and moderate hearing loss within the total otitis media–attributable burden.

Temporal trends were evaluated using annual percentage change (APC), estimated by log-linear regression of age-standardised prevalence on calendar year. Countries classified as aggregated regions in the GBD hierarchy were excluded from APC analyses. APC estimates with 95% confidence intervals crossing zero were interpreted as stable trends.

A decomposition analysis quantified the absolute contributions of population growth, population ageing, and changes in age-specific YLD rates to overall differences in YLDs between 1990 and 2023, using a stepwise counterfactual approach. Countries were ranked according to age-standardised prevalence in 2023. Ninety-five percent uncertainty intervals (UIs), derived from GBD posterior sampling, are reported in the Supplementary Materials.

All analyses were conducted in R version 4.3.2 using the tidyverse, janitor, broom, and ggplot2 packages. As this study used only publicly available, aggregated data from the GBD 2023 Results Tool and involved no individual-level or identifiable information, ethics approval and informed consent were not required.

## Results

**Fig 1:**
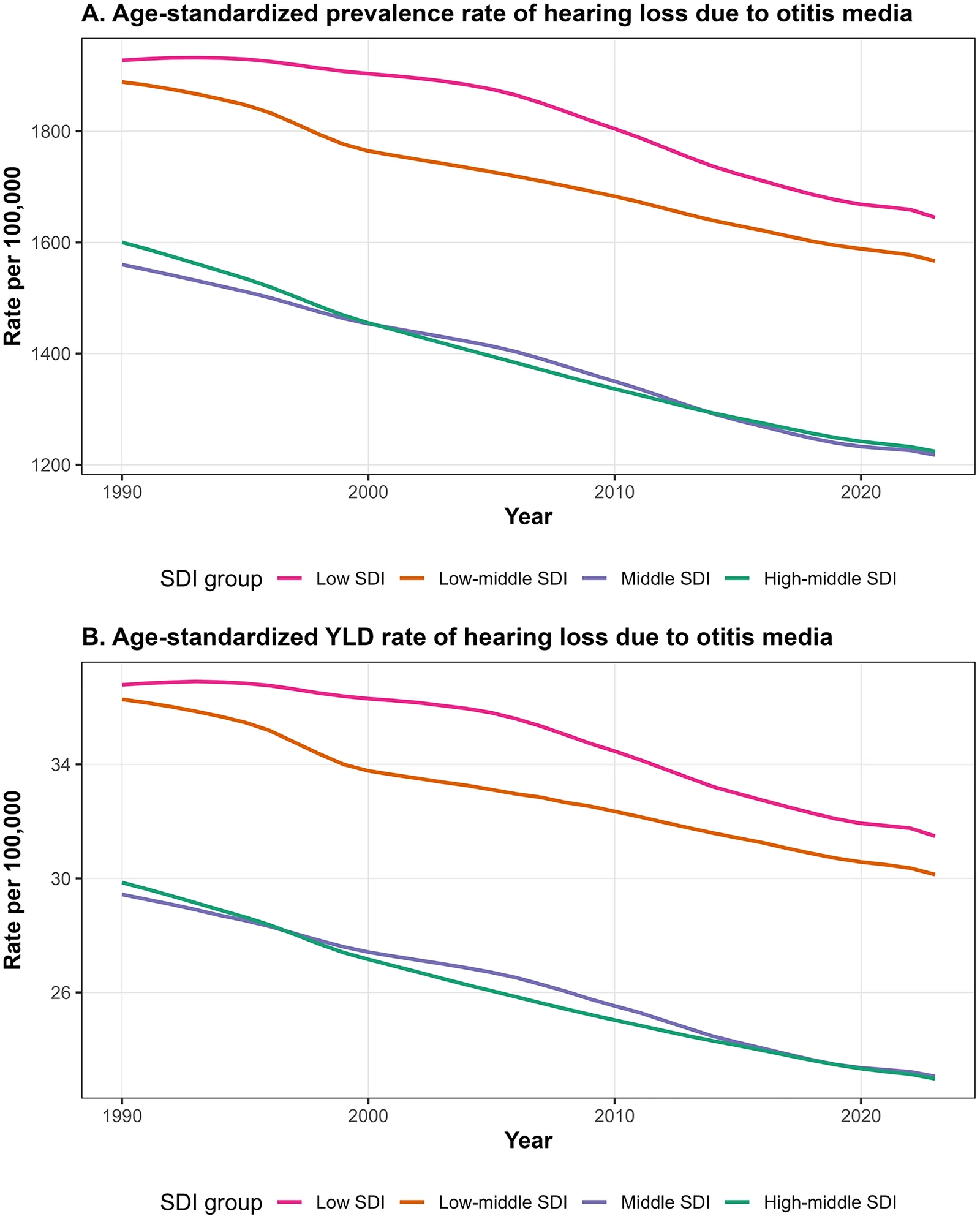
Age-standardised prevalence and YLD rates of hearing loss attributable to otitis media by Socio-demographic Index (SDI) group, 1990–2023. Panel A shows trends in age-standardised prevalence across four SDI groups (low, low-middle, middle, high-middle) from 1990 to 2023. Panel B presents corresponding age-standardised YLD rates. Rates are per 100 000 population and represent GBD 2023 point estimates. Ninety-five percent uncertainty intervals (UIs) are omitted for visual clarity but available in Supplementary Materials. SDI = Socio-demographic Index; YLD = years lived with disability.

Globally, the age-standardised prevalence of hearing loss attributable to otitis media declined modestly between 1990 and 2023, with clear heterogeneity across SDI groups (Fig 1; Table 1). High-middle SDI settings consistently had the lowest prevalence throughout the period, whereas low- and low-middle SDI settings sustained substantially higher burdens with only limited improvement. Trends in age-standardised YLDs mirrored these patterns, with persistent disparities by development level.

**Table 1:** Global and SDI-level age-standardised prevalence and YLDs of hearing loss attributable to otitis media in 1990, 2000, and 2023.

SDI group	Prevalence 1990	Prevalence 2000	Prevalence 2023	Δ Prevalence 1990–2023 (%)	YLDs 1990	YLDs 2000	YLDs 2023	Δ YLDs 1990–2023 (%)
Global	1493 (1261–1757)	1427 (1207–1682)	1275 (1083–1512)	−218 (−15)	28 (17–47)	27 (16–45)	24 (14–40)	−4 (−14)
Low SDI	1927 (1568–2300)	1903 (1549–2275)	1645 (1364–1966)	−282 (−15)	37 (22–61)	36 (22–60)	31 (18–52)	−5 (−14)
Low-middle SDI	1889 (1562–2230)	1764 (1472–2076)	1567 (1319–1869)	−322 (−17)	36 (22–60)	34 (20–57)	30 (18–50)	−6 (−17)
Middle SDI	1560 (1303–1829)	1454 (1218–1722)	1218 (1021–1444)	−342 (−22)	29 (17–50)	27 (16–46)	23 (13–39)	−6 (−22)
High-middle SDI	1600 (1347–1886)	1455 (1233–1715)	1224 (1036–1443)	−376 (−24)	30 (18–51)	27 (16–46)	23 (13–39)	−7 (−23)

Values are age-standardised rates per 100,000 population and are presented as estimates with 95% uncertainty intervals (UIs). Percentage change refers to relative change between 1990 and 2023. Statistical comparisons were interpreted based on overlap of 95% uncertainty intervals derived from GBD posterior distributions rather than conventional P values.

**Fig 2:**
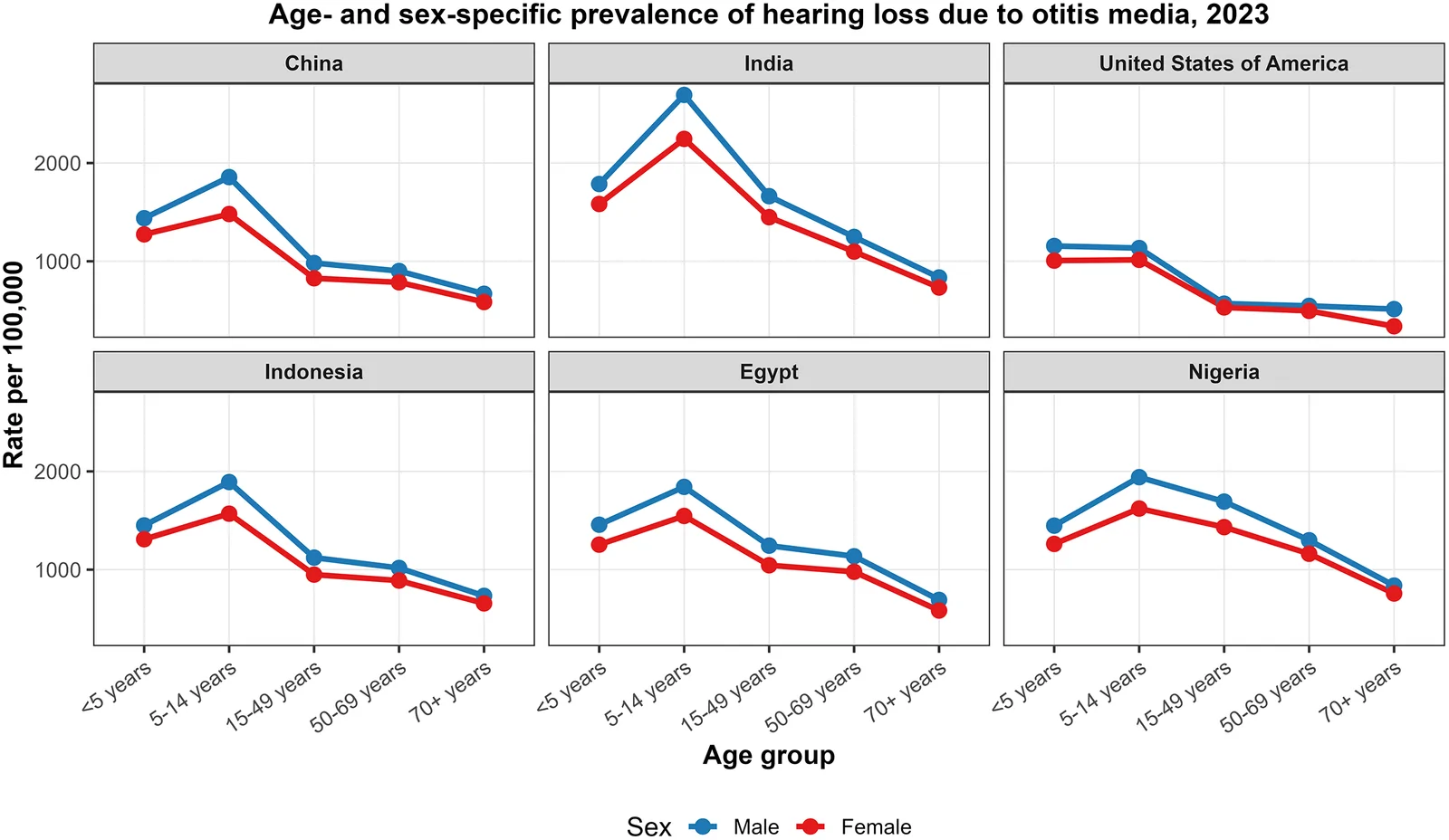
Age- and sex-specific prevalence of hearing loss attributable to otitis media in six high-burden countries, 2023. Prevalence per 100 000 population is shown across age groups (<5, 5–14, 15–49, 50–69, ≥ 70 years) for China, India, the United States, Indonesia, Egypt, and Nigeria. Estimates represent GBD 2023 point values stratified by sex. UIs are available in Supplementary Materials. Countries are listed alphabetically.

Age-specific patterns in 2023 were broadly similar across the six populous and regionally representative countries analysed (Fig 2). Prevalence peaked among children aged 5–14 years and declined steadily with increasing age, reaching its lowest levels among adults aged ≥70 years. Sex differences were evident: males had higher prevalence than females across almost all age groups and countries, consistent with global sex-specific estimates (Table 2).

**Table 2:** Sex differences in age-standardised prevalence of hearing loss attributable to otitis media in 2023.

Location/ SDI group	Male (95% UI)	Female (95% UI)	Male-to-female ratio
Global	1365 (1159–1611)	1183 (1003–1406)	1.15
China	1168 (990–1375)	980 (837–1152)	1.19
India	1746 (1482–2060)	1509 (1273–1808)	1.16
Low SDI	1755 (1450–2092)	1535 (1274–1838)	1.14
Low-middle SDI	1681 (1411–1995)	1449 (1219–1741)	1.16
Middle SDI	1311 (1104–1550)	1121 (935–1325)	1.17
High-middle SDI	1317 (1114–1555)	1127 (956–1333)	1.17
United States	734 (635–850)	661 (565–764)	1.11

Values are age-standardised prevalence rates per 100,000 population in 2023 and are presented as estimates with 95% uncertainty intervals (UIs). Male-to-female ratios greater than 1 indicate higher prevalence among males. Differences between male and female estimates were assessed using uncertainty intervals. Globally, mild hearing loss accounted for most of the age-standardised prevalence attributable to otitis media in 2023, contributing nearly four-fifths of the combined mild-and-moderate burden, with moderate hearing loss comprising the remainder (S1 Table). Temporal patterns from 1990 to 2023 indicated broadly declining trends across countries rather than divergence (Table 3). No country exhibited a statistically significant increase in APC in age-standardised prevalence. Several middle- and high-middle SDI settings experienced the steepest declines, with APC values of approximately –1% to –3% per year. Most other countries showed modest declines or stable trends.

**Table 3:** Ten countries with the largest decreases in age-standardised prevalence of hearing loss attributable to otitis media, 1990–2023.

Rank	Country	APC (95% CI)	Trend
1	China	−1.34 (−1.38 to −1.31)	Decreasing
2	Bangladesh	−0.85 (−0.98 to −0.72)	Decreasing
3	Nigeria	−0.80 (−0.95 to −0.64)	Decreasing
4	Ethiopia	−0.75 (−0.89 to −0.62)	Decreasing
5	Indonesia	−0.67 (−0.73 to −0.61)	Decreasing
6	India	−0.65 (−0.71 to −0.60)	Decreasing
7	Egypt	−0.62 (−0.64 to −0.60)	Decreasing
8	Nepal	−0.56 (−0.62 to −0.51)	Decreasing
9	Philippines	−0.49 (−0.53 to −0.44)	Decreasing
10	Iran	−0.49 (−0.56 to −0.42)	Decreasing

**Fig 3:**
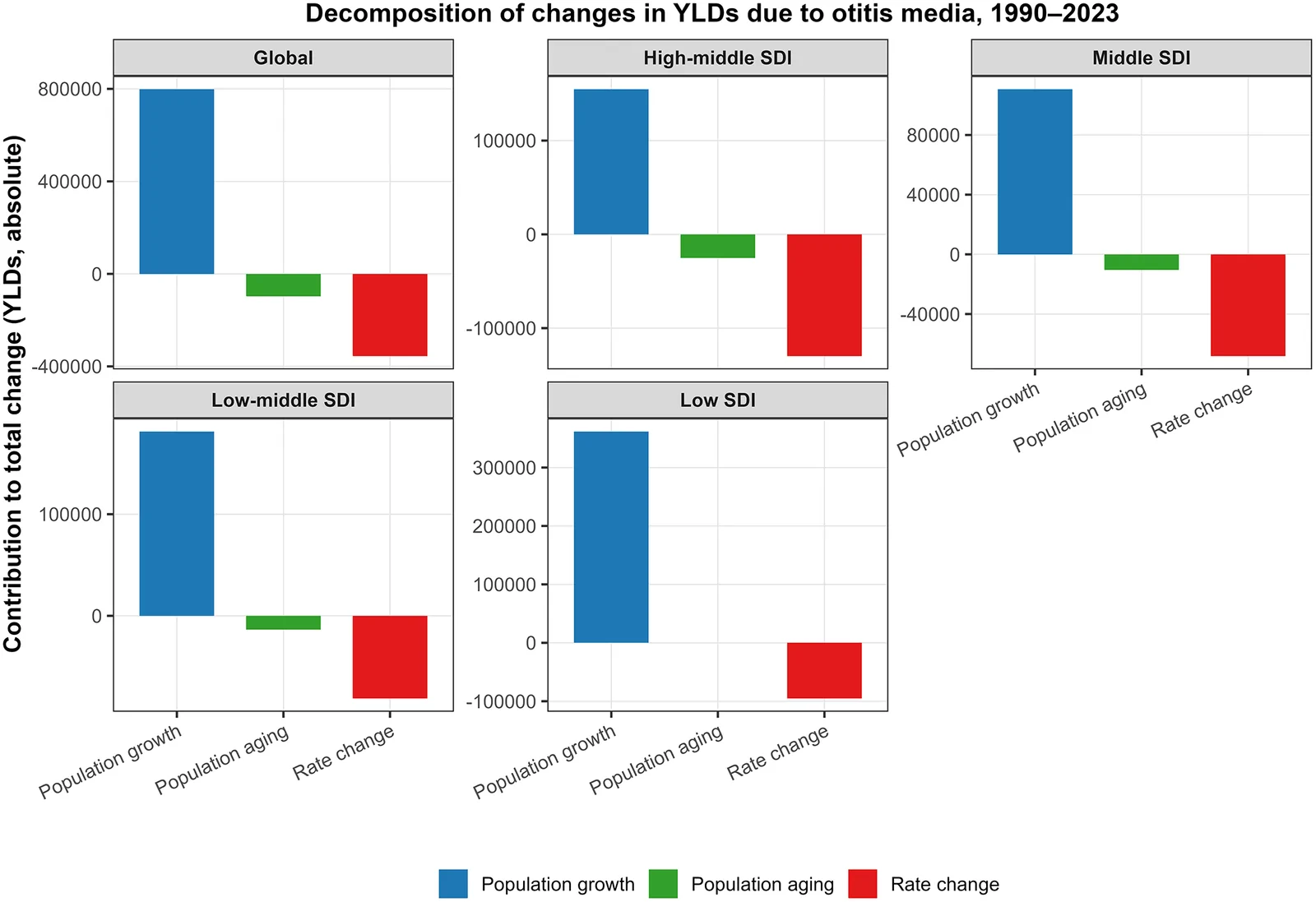
Decomposition of changes in years lived with disability (YLDs) due to otitis media at the global level and by SDI group, 1990–2023. The figure presents the contributions of population growth, population ageing, and changes in age-specific YLD rates to total YLD change from 1990 to 2023. Positive values indicate increases; negative values reflect reductions due to declines in age-specific rates. SDI = Socio-demographic Index; YLD = years lived with disability. Among the countries included in the country-level analysis, the highest age-standardised prevalence in 2023 was observed in several countries, including Nepal, Bangladesh, India, Ethiopia, Nigeria, and Kenya, whereas China, Mexico, Colombia, Russia, Japan, and the United States remained among the lowest-burden countries (S2 Table). Disparities by age, sex, and development level persisted throughout the 1990–2023 period.

APC indicates annual percentage change estimated from log-linear regression of age-standardised prevalence between 1990 and 2023. Countries with 95% confidence intervals entirely below zero were classified as showing decreasing trends. Decomposition analysis revealed substantial differences across SDI groups and at the global level in the drivers of YLD change between 1990 and 2023 (Fig 3). In low- and low-middle SDI settings, population growth was the principal contributor to rising YLDs, with additional, though smaller, contributions from population ageing. In contrast, in middle- and high-middle SDI settings, reductions in age-specific YLD rates generated sizeable negative contributions that partially counteracted demographic pressures. At the global level, population growth accounted for the largest increase in YLDs, while declining age-specific rates mitigated part of this rise.

## Discussion

### Principal findings

This study provides an updatedassessment of hearing loss attributable to otitis media at the global level, across SDI groups, and among selected countries. Although global age-standardised prevalence and YLD rates declined modestly over the 34-year period, progress was uneven. Low- and low-middle SDI settings continued to experience the highest burdens, with far slower improvements than those observed in middle- and high-middle SDI regions. Age-specific patterns were consistent across countries, with prevalence peaking among children aged 5–14 years and declining thereafter. Males had higher prevalence than females across most settings, mirroring global sex-specific estimates. Higher prevalence among males may reflect differences in environmental exposure, healthcare-seeking behaviour, and potential biological susceptibility. However, these mechanisms cannot be directly evaluated using GBD estimates. Mild hearing loss accounted for nearly four-fifths of the global age-standardised burden, with moderate hearing loss comprising the remainder. APC declined in all countries, and no country exhibited a statistically significant increasing trend in age-standardised prevalence between 1990 and 2023. Decomposition analyses showed that population growth was the primary driver of rising YLDs in low- and low-middle SDI settings, whereas declining age-specific YLD rates partially offset demographic pressures in middle- and high-middle SDI regions. The highest 2023 prevalence was observed in Bangladesh, India, Ethiopia, Nigeria, and Kenya, while China, Mexico, Colombia, Russia, Japan, and the United States had the lowest estimated burdens.

### Interpretation in context of existing evidence

Our findings are consistent with earlier analyses showing that otitis media remains one of the leading causes of preventable hearing loss globally and disproportionately affects children in lower-SDI settings [14–16]. This study extends previous work in several important ways, building on earlier GBD iterations [2]. First, the concentration of peak prevalence among children aged 5–14 years—rather than exclusively among children under five—highlights the substantial contribution of school-age otitis media sequelae to long-term auditory outcomes, underscoring the need for targeted prevention strategies among school-aged children [17,18]. Second, the uniform downward APC across countries contrasts with patterns observed in earlier decades, when several regions showed increasing prevalence. This shift suggests broad gains related to improvements in vaccination, infection control, and child health services [19–21]. Third, the predominance of mild hearing loss underscores a substantial “hidden” burden that remains largely unaddressed within health systems, where detection often prioritises moderate or severe cases [22]. Finally, the decomposition analysis provides new insights into the interaction between epidemiological progress and demographic change. In many low-resource settings, population growth continues to outpace declines in age-specific YLD rates, sustaining or increasing overall disability burdens [23].

### Strengths of the study

This study has several strengths. It uses the updated GBD 2023 framework, enabling consistent comparisons across 34 years at the global level, across SDI groups, and among selected countries. The integration of age-standardised, age-specific, and sex-specific metrics provides a more detailed and nuanced understanding of the burden of otitis media–attributable hearing loss. The inclusion of severity composition, national APC trajectories, and decomposition analysis adds depth by capturing the epidemiological, demographic, and health-system factors shaping global patterns. Harmonisation of location names and consistent categorisation of SDI groups further support valid cross-country comparisons.

### Limitations

Several limitations should be acknowledged. First, all estimates depend on the modelling assumptions and input data of the GBD 2023 study, which remain sparse in many low-resource and hard-to-reach settings, particularly for population-based hearing assessments [6]. Although uncertainty intervals incorporate sampling and modelling variability, they cannot fully compensate for gaps in primary data availability. Second, severity classifications and age-specific YLD estimates rely on available audiometric evidence and may underdetect fluctuating or subclinical hearing loss associated with otitis media. Third, while decomposition analysis quantifies the contributions of demographic and epidemiological factors to YLD change, it cannot attribute causality to specific interventions such as vaccination, antimicrobial treatment, or early detection programmes. Fourth, residual heterogeneity may persist because of differences in diagnostic practices, clinical coding, and reporting standards across countries. Finally, YLDs represent population-level health loss quantified using disability weights within the GBD framework and should not be interpreted as equivalent to clinical disability in every individual patient. The restriction to mild and moderate hearing loss reflects limitations of the current GBD severity-mapping framework rather than the absence of clinically severe outcomes, as otitis media predominantly causes mild-to-moderate conductive hearing loss [24], and GBD-based analyses show that severe or profound hearing loss represents a small proportion of cases across etiologies [2,25].

Although GBD provides standardized estimates enabling international comparisons, differences in data availability, diagnostic practices, and healthcare systems may influence country-level estimates, and findings should therefore be interpreted cautiously.

### Implications for policy and practice

These findings carry several policy implications. The high burden in low- and low-middle SDI settings underscores the urgency of expanding access to timely management of acute and chronic otitis media—including affordable antimicrobials, diagnostic capacity, referral pathways, and early preventive care—particularly for school-aged children [3,4,26,27]. The pronounced peak in school-age groups highlights the importance of incorporating ear and hearing screening into preschool and school health programmes [16,28,29]. The predominance of mild hearing loss further emphasises the need for detection strategies that extend beyond approaches targeting only moderate or severe impairment [1,21,30,31]. Demographic pressures—especially rapid population growth in regions with large child populations—suggest that disability from otitis media will continue to rise unless early detection, prevention, and treatment are substantially scaled, placing additional strain on already stretched health systems. Strengthening primary-level ear and hearing care, integrating services into universal health coverage, and prioritising interventions in high-burden countries could substantially reduce long-term disability, especially by improving early detection, timely treatment, and preventive strategies in high-risk populations.

## Conclusions

Hearing loss attributable to otitis media remains a substantial and unevenly distributed global health burden worldwide. Despite modest overall declines since 1990, large and persistent disparities by age, sex, and SDI continue to impede progress. Children in low-SDI settings bear the greatest burden, while older adults also experience considerable disability due to demographic change, particularly in regions with ageing populations. These findings highlight the need to prioritise early detection and management of otitis media; expand access to ear and hearing care within primary health systems; integrate school-based hearing screening; and strengthen preventive strategies such as vaccination. Targeted investments in underserved populations, especially in high-burden low-SDI settings, will be essential to reduce avoidable disability and narrow the inequities identified in this study.

## Supporting information

S1 Table Severity composition of age-standardized prevalence of hearing loss attributable to otitis media, Global, 2023. (DOCX)

S2 Table Age-standardized prevalence of hearing loss attributable to otitis media among countries included in the country-level analysis, 2023. (DOCX)

S3 Table Age-standardized prevalence of hearing loss attributable to otitis media by Socio-demographic Index (SDI) group, 1990–2023. (DOCX)

S4 Table Age-specific prevalence of hearing loss attributable to otitis media, Global, 2023. (DOCX)
