# Supplementary material for: Global patterns and demographic drivers of hearing loss attributable to otitis media, 1990–2023
Source: PLoS One. 2026 Aug 28;21(8):e0356787. doi: 10.1371/journal.pone.0356787 (PMC13524236; doi:10.1371/journal.pone.0356787)
Supplement: Supplementary file 1 [file pone.0356787.s001.docx]

Supplementary Table S1. Severity composition of age-standardized prevalence of hearing loss attributable to otitis media, Global, 2023

| Severity | Proportion (%) |
| --- | --- |
| Mild hearing loss | 78.1 |
| Moderate hearing loss | 21.9 |

Table note: Proportions were derived from Global Burden of Disease (GBD) 2023 estimates. Only mild and moderate severity categories are defined within the current GBD severity-mapping framework.
