# Supplementary material for: Global patterns and demographic drivers of hearing loss attributable to otitis media, 1990–2023
Source: PLoS One. 2026 Aug 28;21(8):e0356787. doi: 10.1371/journal.pone.0356787 (PMC13524236; doi:10.1371/journal.pone.0356787)
Supplement: Supplementary file 2 [file pone.0356787.s002.docx]

Supplementary Table S2. Age-standardized prevalence of hearing loss attributable to otitis media among countries included in the country-level analysis, 2023

| Country | Age-standardized prevalence rate (per 100,000) | Lower 95% UI | Upper 95% UI | Rank |
| --- | --- | --- | --- | --- |
| Nepal | 1918.14244860301 | 1595.28828512247 | 2258.92661461138 | 1 |
| Bangladesh | 1770.46372823617 | 1455.06593517341 | 2094.22612611731 | 2 |
| India | 1630.95260472126 | 1374.66006913808 | 1951.69303113564 | 3 |
| Ethiopia | 1539.53772058632 | 1285.86729271421 | 1823.33501818691 | 4 |
| Nigeria | 1444.51268337361 | 1194.03702910452 | 1737.62517130466 | 5 |
| Kenya | 1346.71866529038 | 1124.37832972503 | 1601.30881398795 | 6 |
| Ukraine | 1305.54556208565 | 1101.08920057632 | 1548.96765105239 | 7 |
| Philippines | 1241.1121302429 | 1047.43971259459 | 1477.80122303623 | 8 |
| Iran | 1236.48579732863 | 1054.26595090051 | 1456.42605776855 | 9 |
| Egypt | 1223.31057635631 | 999.5774333956 | 1453.24837944993 | 10 |
| Indonesia | 1173.02124860779 | 989.3304020299 | 1392.13477366275 | 11 |
| Mexico | 1144.18506930152 | 981.1353352702 | 1339.34319090869 | 12 |
| Colombia | 1139.49331938912 | 962.72225215889 | 1358.31243514566 | 13 |
| Russia | 1103.86905994817 | 938.5222488602 | 1290.97713998944 | 14 |
| China | 1078.97028692443 | 917.97882654159 | 1263.22087014405 | 15 |
| Japan | 785.64602619413 | 668.59714065682 | 915.42633112691 | 16 |
| United States of America | 697.36462131056 | 598.01199129422 | 803.0077102095 | 17 |

Table note: Values are age-standardized prevalence rates per 100,000 population with 95% uncertainty intervals (UIs). Countries are limited to locations available in the country-level analysis dataset.
