# Supplementary material for: Global patterns and demographic drivers of hearing loss attributable to otitis media, 1990–2023
Source: PLoS One. 2026 Aug 28;21(8):e0356787. doi: 10.1371/journal.pone.0356787 (PMC13524236; doi:10.1371/journal.pone.0356787)
Supplement: Supplementary file 3 [file pone.0356787.s003.docx]

Supplementary Table S3. Age-standardized prevalence and YLD rates of hearing loss attributable to otitis media by Socio-demographic Index (SDI) group, 1990–2023.

| Year | Low SDI | Low-middle SDI | Middle SDI | High-middle SDI |
| --- | --- | --- | --- | --- |
| 1990.0 | 1927.48623834136 | 1888.6513995574 | 1560.0592243604 | 1600.17853478186 |
| 2000.0 | 1903.46206364705 | 1764.41647835196 | 1453.5928460535 | 1455.13095801572 |
| 2010.0 | 1804.36076497701 | 1682.89884967828 | 1350.04629017028 | 1336.42750507226 |
| 2023.0 | 1645.05890450611 | 1566.5891664982 | 1217.62952216763 | 1224.25181381759 |

Table note: Values represent age-standardized prevalence rates per 100,000 population by SDI group derived from GBD 2023 estimates.
