# Supplementary material for: Global patterns and demographic drivers of hearing loss attributable to otitis media, 1990–2023
Source: PLoS One. 2026 Aug 28;21(8):e0356787. doi: 10.1371/journal.pone.0356787 (PMC13524236; doi:10.1371/journal.pone.0356787)
Supplement: Supplementary file 4 [file pone.0356787.s004.docx]

Supplementary Table S4. Age-specific prevalence of hearing loss attributable to otitis media, Global, 2023

| Age group | Age-specific prevalence rate (per 100,000) | Lower 95% UI | Upper 95% UI |
| --- | --- | --- | --- |
| <5 years | 1492.88248776611 | 1347.62149501951 | 1638.11923246916 |
| <5 years | 1327.77687837807 | 1195.60204954436 | 1462.36430224753 |
| <5 years | 1412.8443751951 | 1274.41225154863 | 1559.24901883102 |
| 5-14 years | 2013.21086576107 | 1642.28322980837 | 2369.59116065266 |
| 5-14 years | 1699.04852592746 | 1388.66104524819 | 2003.11214049592 |
| 5-14 years | 1860.85531942913 | 1520.43173751061 | 2189.6655098749 |
| 15-49 years | 1287.85220877069 | 951.48774478869 | 1665.71919019702 |
| 15-49 years | 1122.32946286787 | 833.00889299493 | 1449.74613144562 |
| 15-49 years | 1206.29330961424 | 891.88045107171 | 1559.05209771559 |
| 50-69 years | 999.43220221654 | 659.51650190534 | 1355.78745366044 |
| 50-69 years | 877.80305841954 | 576.57264116403 | 1233.37909380276 |
| 50-69 years | 937.57655218255 | 613.31345792432 | 1295.00474651848 |
| 70+ years | 678.4930910835 | 463.84376646176 | 963.55489643512 |
| 70+ years | 553.9117208102 | 376.30221580187 | 787.96678209948 |
| 70+ years | 608.94149368792 | 415.4058765259 | 868.53885579097 |

Table note: Values represent age-specific prevalence rates per 100,000 population with 95% uncertainty intervals (UIs) derived from GBD 2023 estimates.
