# Supplementary material for: Global patterns and demographic drivers of hearing loss attributable to otitis media, 1990–2023
Source: PLoS One. 2026 Aug 28;21(8):e0356787. doi: 10.1371/journal.pone.0356787 (PMC13524236; doi:10.1371/journal.pone.0356787)
Supplement: Supplementary file 5 [file pone.0356787.s006.docx]

Dear Editor and Reviewers,

We sincerely thank the Academic Editor and the reviewers for their careful evaluation of our manuscript and for providing valuable and constructive comments. We greatly appreciate the time and effort invested in reviewing our work.

We have carefully considered all comments and revised the manuscript accordingly. The revisions have improved the clarity, presentation, and interpretation of our findings. Below, we provide point-by-point responses to each comment. All changes have been incorporated into the revised manuscript, and modifications are highlighted in the tracked-change version.

Response to Reviewer #1

Comment 1: The supplementary Table S1 and S2 were not comprehensive. I suspect that some data was missing from the tables.

Response: Thank you for this important comment. We have carefully reviewed the Supplementary Tables and clarified the scope of the country-level analyses in the revised manuscript. The revised manuscript now explicitly distinguishes between analyses conducted at the global level, SDI-group level, and country level. Global and SDI-level estimates were analysed using aggregated GBD locations, whereas country-level analyses were restricted to locations available in the downloaded dataset. The revised manuscript no longer implies that country-level analyses covered all GBD locations, and the scope of the country-level estimates has been clarified throughout the manuscript. We have also revised the Supplementary Information to improve transparency and completeness: Supplementary Table S1 has been clarified to present the global severity distribution of mild and moderate hearing loss attributable to otitis media in 2023. Supplementary Table S2 has been revised to present age-standardized prevalence estimates for all countries included in the country-level analysis. We have also clarified in the Methods section that country-level analyses were restricted to locations available in the downloaded dataset. These revisions clarify the data sources and analytical scope of the study.

Comment 2: Values in Table 1 and 2 have too many decimal. No decimals are needed, only whole values.

Response: Thank you for this suggestion. We have revised Tables 1 and 2 by rounding all estimates and uncertainty intervals to whole numbers, improving readability while maintaining the accuracy required for interpretation.

Comment 3: p-values are missing in the Tables 1 and 2.

Response: Thank you for raising this point. Because this study is based on GBD estimates with posterior uncertainty intervals rather than primary observational data, conventional hypothesis-testing P values are not routinely used for comparisons. Therefore, we considered uncertainty intervals more appropriate for interpretation of GBD estimates. To clarify this issue, we have added explanatory notes to Tables 1 and 2 indicating that comparisons were interpreted based on overlap of 95% uncertainty intervals derived from GBD posterior distributions rather than conventional P values.

Comment 4: Figure 3 is not needed, the same information can be provided in one sentence.

Response: We appreciate this suggestion. Following the reviewer’s recommendation, we removed the original Figure 3 showing severity composition. The corresponding information remains available in the Results section and Supplementary Table S1.

Comment 5: Figure 4 and Table 3 give the identical information. Only one is needed.

Response: Thank you for this helpful suggestion. We agree that the original Figure 4 (APC forest plot) and Table 3 provided overlapping information. Therefore, we removed Figure 4 and retained Table 3, which provides the APC estimates and confidence intervals in a more concise format. This change reduced redundancy and improved manuscript readability. The remaining figure numbers have been updated accordingly.

Comment 6: References to Tables, Figures, and Supplementary data should not be in the Discussion section.

Response: Thank you for this comment. We have carefully reviewed the Discussion section and removed references to specific Tables, Figures, and Supplementary Tables. The Discussion now focuses on interpretation of the findings rather than repeating references to the presented results.

Response to Reviewer #2

Comment 1: The SDI categories have few broad categories. Is that a standard format or its incorporated for the study is not clear. If it is only for the study how valid the categories are? How did the classification come from? The base for categorization should be clear.

Response: Thank you for this important comment. We have clarified the basis of SDI categorization in the Methods section. Specifically, we added that SDI categories followed the standard classification provided by the Institute for Health Metrics and Evaluation (IHME) GBD 2023 framework, including low, low-middle, middle, and high-middle SDI groups. This clarification indicates that the categories were predefined within the GBD framework rather than developed specifically for this study.

Comment 2: The YLD category is wisely used and clearly describes the data on the years of underlying problem. But when mentioning it as disability, question arises can we call it as a disability? Even if the problem is mild category? How this can be justified?

Response: Thank you for raising this important conceptual point. We agree that YLDs in the GBD framework represent population-level health loss rather than clinical disability experienced by every individual. We have revised the Limitations section to clarify that YLDs represent health loss quantified using disability weights within the GBD framework and should not be interpreted as equivalent to clinical disability in every individual patient.

Comment 3: In the prevalence of hearing loss the broad category of 5–14 years have mentioned. Can it be narrowed down to two categories?

Response: Thank you for this suggestion. The age categories used in this study follow the predefined GBD age structure. The downloaded GBD dataset provides estimates according to these standardized age groups, and further subdivision of the 5–14 years category was therefore not possible within the current analytical framework. We have added this clarification to the Methods section.

Comment 4: As the current research considers data globally, generalizing the data requires good justifications.

Response: Thank you for this thoughtful comment. We agree that interpretation of global estimates requires caution. We have added a statement in the Limitations section emphasizing that although GBD provides standardized estimates enabling international comparisons, differences in data availability, diagnostic practices, and healthcare systems may influence country-level estimates. This revision highlights the importance of cautious interpretation of the findings.

Comment 5: What is the reason for males having more prevalence than females? Can it have anything to do with physiological mechanism in auditory system or exposure which can be environmental variables?

Response: Thank you for this valuable suggestion. We have expanded the Discussion section to provide possible explanations for the observed sex differences. Specifically, we noted that higher prevalence among males may reflect differences in environmental exposure, healthcare-seeking behaviour, and potential biological susceptibility. We also clarified that these mechanisms cannot be directly evaluated using GBD estimates and require further investigation.

Comment 6: The strength and limitations are clearly mentioned and I appreciate the authors for providing these inputs in the article.

Response: We sincerely thank the reviewer for this positive comment. We appreciate the recognition of the strengths and limitations of our study.

Comment 7: The graphs and their representations are very clear. Well written conclusion with clear references.

Response: We sincerely thank the reviewer for this encouraging comment.

Additional Editorial Requirements

Requirement 1: Please ensure that your manuscript meets PLOS ONE style requirements.

Response: Thank you for this reminder. We have revised the manuscript according to PLOS ONE formatting requirements, including: title page formatting; corresponding author information; removal of unnecessary address information; consistency of figure and table formatting.

Requirement 2: Please include captions for Supporting Information files at the end of your manuscript.

Response: Thank you. We have added a dedicated “Supporting information” section at the end of the manuscript and updated the corresponding Supplementary Table references accordingly.

Requirement 3: Recommendations regarding citation of previously published works.

Response: We have reviewed the suggested references and considered their relevance to our manuscript. Relevant references have been retained or incorporated where appropriate. No additional citations were added when they were not directly relevant to the scope of this study.

Closing statement

We sincerely appreciate the valuable comments provided by the Editor and Reviewers. We believe that the revisions have substantially improved the clarity and quality of our manuscript, and we hope that the revised version will now be suitable for publication in PLOS ONE.

Sincerely,

Yubin Xue

Corresponding Author
