# Supplementary material for: Global patterns and demographic drivers of hearing loss attributable to otitis media, 1990–2023
Source: PLoS One. 2026 Aug 28;21(8):e0356787. doi: 10.1371/journal.pone.0356787 (PMC13524236; doi:10.1371/journal.pone.0356787)
Supplement: Peer review history

# Peer review: Decision Letter 0

**DOI:** 10.1371/journal.pone.0356787.r001

**Authors:** Rohit Ravi

12 Jul 2026

Dear Dr. Xue,

Thank you for submitting your manuscript to PLOS ONE. After careful consideration, we feel that it has merit but does not fully meet PLOS ONE’s publication criteria as it currently stands. Therefore, we invite you to submit a revised version of the manuscript that addresses the points raised during the review process.

Please submit your revised manuscript by Aug 26 2026 11:59PM. If you will need more time than this to complete your revisions, please reply to this message or contact the journal office at plosone@plos.org. When you're ready to submit your revision, log on to https://www.editorialmanager.com/pone/ and select the 'Submissions Needing Revision' folder to locate your manuscript file.

- A letter that responds to each point raised by the academic editor and reviewer(s). You should upload this letter as a separate file labeled 'Response to Reviewers'.
- A marked-up copy of your manuscript that highlights changes made to the original version. You should upload this as a separate file labeled 'Revised Manuscript with Track Changes'.
- An unmarked version of your revised paper without tracked changes. You should upload this as a separate file labeled 'Manuscript'.

If you would like to make changes to your financial disclosure, please include your updated statement in your cover letter. Guidelines for resubmitting your figure files are available below the reviewer comments at the end of this letter.

If applicable, we recommend that you deposit your laboratory protocols in protocols.io to enhance the reproducibility of your results. Protocols.io assigns your protocol its own identifier (DOI) so that it can be cited independently in the future. For instructions see: https://journals.plos.org/plosone/s/submission-guidelines#loc-laboratory-protocols. Additionally, PLOS ONE offers an option for publishing peer-reviewed Lab Protocol articles, which describe protocols hosted on protocols.io. Read more information on sharing protocols at https://plos.org/protocols?utm_medium=editorial-email&utm_source=authorletters&utm_campaign=protocols.

As the corresponding author, your ORCID iD is verified in the submission system and will appear in the published article. PLOS supports the use of ORCID, and we encourage all coauthors to register for an ORCID iD and use it as well. Please encourage your coauthors to verify their ORCID iD within the submission system before final acceptance, as unverified ORCID iDs will not appear in the published article. *Only*  the individual author can complete the verification step; PLOS staff *cannot*  verify ORCID iDs on behalf of authors.

We look forward to receiving your revised manuscript.

Kind regards,

Rohit Ravi, Ph.D.

Academic Editor

PLOS One

Journal Requirements:

When submitting your revision, we need you to address these additional requirements.

1. Please ensure that your manuscript meets PLOS ONE's style requirements, including those for file naming. The PLOS ONE style templates can be found at

https://journals.plos.org/plosone/s/file?id=wjVg/PLOSOne_formatting_sample_main_body.pdf and

https://journals.plos.org/plosone/s/file?id=ba62/PLOSOne_formatting_sample_title_authors_affiliations.pdf

2.  Please include captions for your Supporting Information files at the end of your manuscript, and update any in-text citations to match accordingly. Please see our Supporting Information guidelines for more information: http://journals.plos.org/plosone/s/supporting-information.

3. If the reviewer comments include a recommendation to cite specific previously published works, please review and evaluate these publications to determine whether they are relevant and should be cited. There is no requirement to cite these works unless the editor has indicated otherwise.

[Note: HTML markup is below. Please do not edit.]

Reviewers' comments:

Reviewer's Responses to Questions

**Comments to the Author**

1. Is the manuscript technically sound, and do the data support the conclusions?

Reviewer #1: No

Reviewer #2: Yes

**********

2. Has the statistical analysis been performed appropriately and rigorously?

Reviewer #1: I Don't Know

Reviewer #2: Yes

**********

3. Have the authors made all data underlying the findings in their manuscript fully available?

The PLOS Data policy

Reviewer #1: No

Reviewer #2: Yes

**********

4. Is the manuscript presented in an intelligible fashion and written in standard English?

Reviewer #1: Yes

Reviewer #2: Yes

**********

Reviewer #1: The supplementary Table S1 and S2 were not comprohensive. I suspect that some data was missing from the tables.

Values in Table 1 and 2 have too many decimal. No decimals are needed, only whole values.

p-values are missing in the Tables 1 and 2.

Figure 3 is not needed, the same information can be provided in one sentence.

Figure 4 and Table 3 give the identical information. Only on is needed.

References to Tables, Figures, and Supplementary data shoud not be in the Discussion seqtion.

Reviewer #2: 1. The SDI categories have few broad categories. Is that a standard format or its incorporated for the study is not clear. If its only for the study how valid the categories are? How did the classification come from? The base for categorization should be clear.

2. The YLD category is wisely used and clearly describes the data on the years of underlying problem. But when mentioning it as disability, question arises can we call it as a disability? Even if the problem is mild category? How this can be justified?

3. In the preavalence of hearing loss the broad category of 5-14yrs have mentioned. Cant it be narrowed down to two categories? Mentioning between 5 to 10yrs and 11 to 14yrs would be good as this would cover as school going children and young adolescents. So the cause and its prevalence would be justified. Also one would get a clear idea about the precautions, the major cause, and frequency of the probem that would arise can be known.

4. As the current research considers data globally, generalizing the data requires good justifications. I appreciate the inclsuion of such huge data and conluding it findings require a lot of effort. However it should be cautious that the generalization also requires a lot of considerations. Hence it should be noted to justify this part in the discussion.

5. What is the reason for males having more prevalence than females is nowhere mentioned as it would roughly gives the involvement of more male samples as a factor. Can it have anything to do with physiological mechanism in auditory system or exposure which can be enviromental variables?

6. The strength and limitations are clearly mentioned and I appreciate the authors for providing these inputs in the article.

7. The graphs and its reprsentations are very clear. Well written conclusion with clear references.

**********

what does this mean?). If published, this will include your full peer review and any attached files.

If you choose “no”, your identity will remain anonymous but your review may still be made public.

**Do you want your identity to be public for this peer review?**  For information about this choice, including consent withdrawal, please see our Privacy Policy

Reviewer #1: No

Reviewer #2: No

**********

[NOTE: If reviewer comments were submitted as an attachment file, they will be attached to this email and accessible via the submission site. Please log into your account, locate the manuscript record, and check for the action link "View Attachments". If this link does not appear, there are no attachment files.]

To ensure your figures meet our technical requirements, please review our figure guidelines: https://journals.plos.org/plosone/s/figures

You may also use PLOS’s free figure tool, NAAS, to help you prepare publication quality figures: https://journals.plos.org/plosone/s/figures#loc-tools-for-figure-preparation.

NAAS will assess whether your figures meet our technical requirements by comparing each figure against our figure specifications.

# Author comment: Author response to Decision Letter 1

**DOI:** 10.1371/journal.pone.0356787.r002

26 Jul 2026

Dear Editor and Reviewers,

We sincerely thank the Academic Editor and reviewers for their careful evaluation of our manuscript and for providing valuable and constructive comments.

We have carefully addressed all comments and revised the manuscript accordingly. The major revisions include clarification of the analytical scope (global, SDI-level, and country-level analyses), improvement of Supplementary Tables, refinement of statistical presentation, removal of redundant figures, clarification of SDI classification and YLD interpretation, and revision of relevant sections in the Methods, Results, Discussion, and formatting according to PLOS ONE requirements.

A detailed point-by-point response to all reviewer comments has been uploaded separately as the file “Response to Reviewers”. A revised manuscript with tracked changes and a clean revised manuscript have also been submitted.

We appreciate the opportunity to revise our manuscript and hope that the revised version is now suitable for publication in PLOS ONE.

Sincerely,

Yubin Xue

Corresponding Author

Attachment Submitted filename: Response to Reviewers.docx

# Editor report: Decision Letter 1

**DOI:** 10.1371/journal.pone.0356787.r003

**Authors:** Rohit Ravi

10 Aug 2026

Global patterns and demographic drivers of hearing loss attributable to otitis media, 1990–2023

PONE-D-26-02699R1

Dear Dr. Xue,

We’re pleased to inform you that your manuscript has been judged scientifically suitable for publication and will be formally accepted for publication once it meets all outstanding technical requirements.

Within one week, you’ll receive an e-mail detailing the required amendments. When these have been addressed, you’ll receive a formal acceptance letter and your manuscript will be scheduled for publication.

An invoice will be generated when your article is formally accepted. Please note, if your institution has a publishing partnership with PLOS and your article meets the relevant criteria, all or part of your publication costs will be covered. Please make sure your user information is up-to-date by logging into Editorial Manager at Editorial Manager® and clicking the ‘Update My Information' link at the top of the page. For questions related to billing, please contact billing support.

If your institution or institutions have a press office, please notify them about your upcoming paper to help maximize its impact. If they’ll be preparing press materials, please inform our press team as soon as possible -- no later than 48 hours after receiving the formal acceptance. Your manuscript will remain under strict press embargo until 2 pm Eastern Time on the date of publication. For more information, please contact onepress@plos.org.

Kind regards,

Rohit Ravi, Ph.D.

Academic Editor

PLOS One

Additional Editor Comments (optional):

The revision is satisfactory.

Reviewers' comments:

# Editor report: Acceptance letter

**DOI:** 10.1371/journal.pone.0356787.r004

**Authors:** Rohit Ravi

PONE-D-26-02699R1

PLOS One

Dear Dr. Xue,

I'm pleased to inform you that your manuscript has been deemed suitable for publication in PLOS One. Congratulations! Your manuscript is now being handed over to our production team.

At this stage, our production department will prepare your paper for publication. This includes ensuring the following:

* All references, tables, and figures are properly cited

* All relevant supporting information is included in the manuscript submission,

* There are no issues that prevent the paper from being properly typeset

You will receive further instructions from the production team, including instructions on how to review your proof when it is ready. Please keep in mind that we are working through a large volume of accepted articles, so please give us a few days to review your paper and let you know the next and final steps.

Lastly, if your institution or institutions have a press office, please let them know about your upcoming paper now to help maximize its impact. If they'll be preparing press materials, please inform our press team within the next 48 hours. Your manuscript will remain under strict press embargo until 2 pm Eastern Time on the date of publication. For more information, please contact onepress@plos.org.

You will receive an invoice from PLOS for your publication fee after your manuscript has reached the completed accept phase. If you receive an email requesting payment before acceptance or for any other service, this may be a phishing scheme. Learn how to identify phishing emails and protect your accounts at https://explore.plos.org/phishing.

If we can help with anything else, please email us at customercare@plos.org.

Thank you for submitting your work to PLOS One and supporting open access.

Kind regards,

PLOS ONE Editorial Office Staff

on behalf of

Dr. Rohit Ravi

Academic Editor

PLOS One
